# Regulation of Endosomal Trafficking by Rab7 and Its Effectors in Neurons: Clues from Charcot–Marie–Tooth 2B Disease

**DOI:** 10.3390/biom13091399

**Published:** 2023-09-16

**Authors:** Ryan J. Mulligan, Bettina Winckler

**Affiliations:** 1 Department of Cell Biology, University of Virginia, Charlottesville, VA 22903, USA; 2 Medical Scientist Training Program, University of Virginia, Charlottesville, VA 22903, USA

**Keywords:** Rab7, neurotrophin, TrkA, endosome biology, neuron, axonal trafficking, Rab effector, Charcot–Marie–tooth 2B

## Abstract

Intracellular endosomal trafficking controls the balance between protein degradation and synthesis, i.e., proteostasis, but also many of the cellular signaling pathways that emanate from activated growth factor receptors after endocytosis. Endosomal trafficking, sorting, and motility are coordinated by the activity of small GTPases, including Rab proteins, whose function as molecular switches direct activity at endosomal membranes through effector proteins. Rab7 is particularly important in the coordination of the degradative functions of the pathway. Rab7 effectors control endosomal maturation and the properties of late endosomal and lysosomal compartments, such as coordination of recycling, motility, and fusion with downstream compartments. The spatiotemporal regulation of endosomal receptor trafficking is particularly challenging in neurons because of their enormous size, their distinct intracellular domains with unique requirements (dendrites vs. axons), and their long lifespans as postmitotic, differentiated cells. In Charcot–Marie–Tooth 2B disease (CMT2B), familial missense mutations in Rab7 cause alterations in GTPase cycling and trafficking, leading to an ulcero-mutilating peripheral neuropathy. The prevailing hypothesis to account for CMT2B pathologies is that CMT2B-associated Rab7 alleles alter endocytic trafficking of the neurotrophin NGF and its receptor TrkA and, thereby, disrupt normal trophic signaling in the peripheral nervous system, but other Rab7-dependent pathways are also impacted. Here, using TrkA as a prototypical endocytic cargo, we review physiologic Rab7 effector interactions and control in neurons. Since neurons are among the largest cells in the body, we place particular emphasis on the temporal and spatial regulation of endosomal sorting and trafficking in neuronal processes. We further discuss the current findings in CMT2B mutant Rab7 models, the impact of mutations on effector interactions or balance, and how this dysregulation may confer disease.

## 1. Introduction

Charcot–Marie–Tooth (CMT) diseases (also known as hereditary motor and sensory neuropathies, or HMSNs) are a group of heterogeneous peripheral neuropathies that often present in adolescence or early adulthood. They are classified into subtypes (CMT1-4, X) by their genetic inheritance pattern, underlying pathophysiology, and clinical presentation, all of which are exceptionally diverse. In total, roughly 60 genetic loci have been linked to all forms of CMT, with varying inheritance patterns [1,2], which collectively are estimated to affect 1 in 2500 individuals [3]. Typical symptomatic features of CMT in patients include distal muscle weakness and wasting, foot deformities like pes cavus, distal sensory deficits, and reduced or absent tendon reflexes [4]. Charcot–Marie–Tooth Type 2B (CMT2B) is a rare autosomal dominant, axonal CMT subtype (CMT2). The CMT2B phenotype is marked by predominant distal sensory loss with normal motor neuron conduction velocity, skin changes, and ulceration and infection complications, which often necessitate digit amputation [5,6]. Genetic loci consistent with CMT2B phenotypes were first identified in a family with ten affected members [7]. Additional families were later identified to carry mutations at the same genetic loci, with similar phenotypic features [6,8,9]. The original classification of the disorder as CMT2B has been debated, as it shares predominant clinical features with hereditary sensory neuropathy type 1 (HSN1) [10]. However, the lack of sensory pain (typical of HSN1) and presence of distal muscle weakness (typical of CMT) have generally led to its acceptance in classification as CMT2B [6]. To this point, there are no current cures for any CMT subtype, and medical treatment is purely symptom management. Molecular genetic studies later revealed that the genetic locus causative for CMT2B encodes the endosomal small GTPase Rab7 and identified two missense mutations (L129F, V162M) associated with the disease [11]. Four additional missense mutations (K126R, K157N, N161T, N161I) have since been identified [11,12,13,14,15]. 

Rabs are a family of small GTPases considered the master controllers of intracellular membrane traffic since they coordinate the behavior of membrane-bound organelles, including endosomes [16,17,18]. Collectively, endosomes facilitate the sorting and trafficking necessary to either degrade or recycle endocytosed cargo [19]. Rab7 functions principally within the degradation arm, and so its activity is integral for controlling the levels of degradative fated cargos, many of which are signaling receptors. Thus, Rab7 activity can modulate the levels of intracellular signaling and signaling duration. This includes the signaling downstream of tyrosine-receptor kinase complexes, like EGF-EGFR [20] or NGF-TrkA [21]. NGF-TrkA provides critical trophic signaling within the peripheral nervous system and is, thus, highly relevant to CMT diseases. 

**Figure 1:**
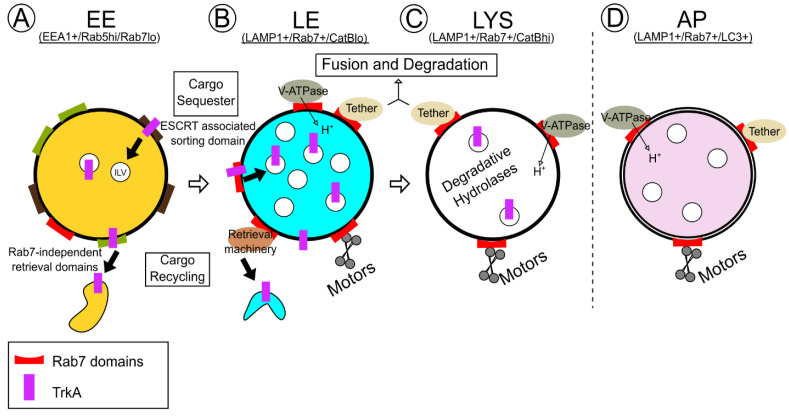
Rab7 executes control over effectors throughout endosomal maturation. Schematic demonstrating endosomal maturation from early endosomes (EEs) to late endosomes (LEs), lysosomes (LYSs), and autophagosomes (APs), each with respective Rab7 levels and domains (red domains). (**A**) EEs are low in Rab7 and have domains specialized in cargo sequestration into intraluminal vesicles (brown domains) and cargo recycling (olive domains), each of which are Rab7-independent. TrkA is displayed as a prototypical cargo (magenta rectangle). The transition from low levels of Rab7 on EEs to high levels on LEs marks a key endosomal maturation step. (**B**) On LEs, Rab7 domains contribute to many functions, including Rab7-dependent TrkA sequestration and recycling (brown oval), acidification via proton pumps (green oval), motility via motors (gray helix), and fusion via tethers (beige oval), all of which contribute to the balance between continued TrkA signaling versus TrkA degradation. (**C**) LYS and (**D**) AP Rab7 functions mirror those on LEs that facilitate cargo degradation.

Specific Rabs are characteristic of particular endosomal compartments and provide unique functions to such compartments through effector proteins [16,17,18]. For example, Rab5 is associated with early endosomes, while Rab7 is associated with late endosomes. In fact, Rab5 to Rab7 transitions are required for early to late endosomal maturation and, subsequently, permit the new association of Rab7 effectors to direct late endosomal behavior [22,23]. On late endosomes, multiple effectors interact with active Rab7 to facilitate a process of late endosomal maturation: late endosomes acquire degradative capacity, motility, acidity, and fusion machinery that makes them functionally more similar to and capable of fusing with lysosomes. Lysosomes, and often autophagosomes, are also Rab7-positive and associated with Rab7 effectors (Figure 1). Ultimately, how Rab7 regulates unique subcellular processes on diverse compartments (ranging from early endosomes to autophagosomes) depends on the particular Rab7 effector choices at each endocytic step.

Endocytic trafficking is especially critical and challenging in neurons. Neurons are spatially complex, highly branched, and exceptionally large with distinct domains (dendrites vs. axons). Further, neurons are post-mitotic terminally differentiated cells and must survive for the lifespan of an organism. Thus, endosomal functions such as Rab7-mediated degradation must be maintained with high fidelity in complex space for extremely long times. It is, therefore, not surprising that mutations in genes encoding proteins related to intracellular trafficking, such as Rab7, are causative for inherited neurologic disease. In fact, both inherited and sporadic neurologic and neurodegenerative disease can often be linked to dysfunction in the endocytic pathway [24]. Rab7 is ubiquitously expressed, and Rab7 knockout mice are early embryonically lethal [25]. CMT2B mutations in Rab7 are compatible with life and have minimal effects on mouse body weight and survival [26]. Instead, they preferentially impact peripheral neurons versus other neuronal or non-neuronal cell types and are, thus, thought to be mild hypo- or hypermorphs. Notably, the mildly dysfunctional CMT2B Rab7 alleles cause pathologies primarily in long axons, consistent with the notion that endocytic traffic in neurons is highly complex and that alterations in long-distance axonal traffic likely contribute to CMT2B disease phenotypes. Therefore, neurons might have less tolerance for even mild Rab7 dysfunction, compared to other cell types. As intracellular trafficking mechanisms, and specifically Rab7 activity, contribute to the control of tyrosine-receptor kinase signaling and degradation, the prevailing hypothesis to explain Rab7-driven CMT2B disease has been an imbalance in trophic signaling, like that of NGF-TrkA [27]. Within this review, we will discuss the molecular behavior and physiologic roles of neuronal Rab7. We will highlight key effector proteins by following the endocytic journey of the TrkA receptor as a prototypical Rab7- and CMT2B-relevant cargo. Finally, we will discuss how mutant Rab7 alters key effector functions throughout neuronal endosomal trafficking and how this may impact neurotrophic signaling within the context of ordered Rab7 effector cascades in space.

## 2. Molecular Basis of Rab7 Function and Disruption in Disease

In order to appreciate how neuronal Rab7 interacts with effector proteins and controls cargo degradation, it is important to first gain an appreciation for its behavior and regulation at the molecular level. Like all Rabs, Rab7 activity is controlled by cycling between an active GTP-bound state and an inactive GDP-bound state. In the active GTP-bound state, Rab7 associates with membranes and binds effector proteins that confer regulatory properties to the membranous compartments it is associated with. GTP/GDP cycling of Rabs is controlled by a balance of guanine exchange factor (GEF) and GTPase-activating protein (GAP) activity. The only currently described Rab7 GEF is the Mon1-Ccz1 complex, which facilitates the exchange of GDP for GTP on Rab7, in conjunction with inactivation of Rab5 [28,29,30]. This facilitates the Rab5 to Rab7 conversion required for early to late endosome maturation [22,23]. Conversely, a multitude of Rab7 GAPs to drive GTP to GDP conversion, particularly of the TBC family of proteins, have been described [28]. 

The six CMT2B-linked missense mutations in Rab7 (K126R, L129F, K157N, N161T, N161I, V162M) show autosomal dominant inheritance. They all map to conserved regions of Rab7, which are essential for nucleotide binding [15,31]. A crystal structure of the Rab7^L129F^ CMT2B mutant protein bound to a non-hydrolyzable GTP analog revealed that the mutation drives protein conformational changes limited to the nucleotide-binding pocket, whereas the switch regions responsible for effector binding mirror that of wild type [32]. In silico modeling also found that residues mutated in CMT2B alleles reside within the GTP-binding pocket and alter hydrogen bond capabilities of amino acid side chains with the nucleotide’s ribose oxygen [15,33]. Mutant Rab7 alleles have an increased nucleotide exchange rate that occurs independently of Mon1-Ccz1 GEF activity [32]. Consequently, Rab7 GTP/GDP binding ratios are increased in mutants, as there is more GTP than GDP in the cell [32,33,34]. As such, CMT2B Rab7 mutants can collectively be thought of as GEF-independent, ‘rapid cycling’ mutants, which are disproportionately GTP bound, leading to increased membrane association. Together, these findings suggest that the downstream effects of CMT2B mutants are a result of increased nucleotide cycling, leading to relative independence from regulated GEF and GAP activities. One common notion is that CMT2B alleles are gain-of-function and bind all Rab7 effectors in a manner that is dysregulated in time and space [31,35]. A previous review discussed the evidence for and against CMT2B alleles being gain of function as opposed to being haploinsufficient [27].

Beyond nucleotide binding, Rab7 activity is also modulated by a number of post-translational modifications, notably phosphorylation, ubiquitination, and palmitoylation, which was adequately reviewed in [36]. The extent to which these other modifications interplay with the Rab7 nucleotide state to control effector binding and activity is an area of active investigation. For instance, phosphomimetic Rab7 mutants (at position S72) are predominantly GDP-bound, while phospho-dead mutants are GTP-bound, suggesting that phosphorylation at S72 might negatively regulate GTP binding [37]. How this is achieved molecularly is unknown. Additionally, two recent reports demonstrate that the Parkin-mediated ubiquitination of Rab7 contributes to increased GTP-Rab7 levels and drives Rab7 more stably onto membranes. This leads to increased colocalization with the lysosomal protein LAMP1 [38,39]. It is wholly unknown how post-translational modifications may be impacted in CMT2B mutants. It would be interesting to explore whether rapid nucleotide cycling (as found in the CMT2B alleles) impacts the ability of Rab7-modulating kinases or ubiquitin ligases to carry out their function, or conversely, if post-translational modifications can modulate nucleotide or effector binding despite rapid cycling.

## 3. Physiologic Control of Rab7 in Neuronal Trafficking and Trophic Signaling

**Figure 2:**
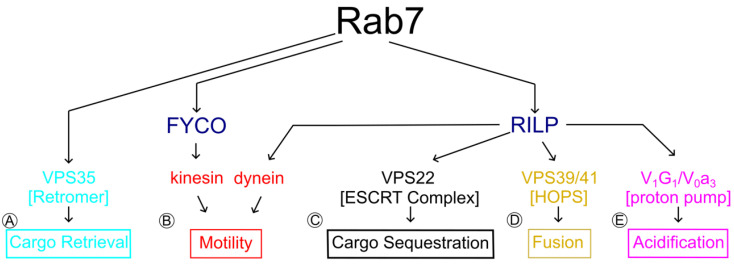
Specific Rab7 effector proteins confer particular properties to endosomes. Schematic demonstrating the breadth and interrelationship of Rab7 effectors and their functions. (**A**) Active Rab7 (top) facilitates retrieval of cargo like TrkA through interactions with Vps35 and the retromer complex (cyan), preserving active TrkA signals. (**B**) Rab7 interacts with the kinesin motor adaptor protein FYVE and coiled-coil (CC) domain containing 1 (FYCO1) (navy) to confer plus-end directed endosome motility, which, in axons, is directed toward the distal axon (red). Rab-interacting lysosomal protein (RILP) is the Rab7-interacting dynein motor adaptor, conferring minus-end (toward the soma) directed motility (red). RILP is also an essential interacting partner for other active Rab7 effector complexes vital for TrkA degradation, including (**C**) VPS22 (ESCRT)-mediated cargo sequestration into intraluminal vesicles (black), (**D**) VPS39/VPS41 (HOPS)-mediated late endosome homo- and hetero-typic fusion with lysosomes (gold), and (**E**) V_1_G_1_/V_0_a_3_ (H^+^-V-ATPase)-mediated regulation of endosomal acidification (magenta). Together, Rab7-dependent motility, acidification, fusion, and proper cargo localization (retrieval vs. sequestration) contribute to TrkA signal duration and degradation in neurons.

Rab7 is important for late endosomal, lysosomal, and autophagosomal functions in all cell types. Early studies in non-neuronal cells demonstrated that Rab7 controls multiple steps along the endo/lysosomal pathway. The earliest Rab7-regulated step is to facilitate the transition from early to late endosome identity. Subsequently, Rab7 controls late endosomal maturation, including trafficking of internalized receptor complexes, motility or positioning of organelles, and fusion with lysosomes [20,40,41,42,43,44,45]. These functions are achieved through interactions of active GTP-Rab7, with an extensive number of effector proteins (Figure 2), some of which we will discuss more in depth in the context of CMT2B in subsequent sections. 

In neurons, the somatic compartment is functionally similar to non-neuronal cells, and Rab7 likely plays similar roles. However, neurons have long axons and highly arborized dendrites, which complicate trafficking processes and demand on Rab7 function. In dendrites, distinct endosomal compartments are found in characteristic spatial distributions, with late endosomes more sparse distally and lysosomes overwhelmingly proximal [46,47,48]. Rab7 populates late endosomal/lysosomal compartments that are more degradative as they localize more proximally in the dendrite and the soma [46,49,50]. Rab7 activity and the dynein-associated motility of Rab7-positive compartments promote the retrograde trafficking of late endosomes and are essential for the degradation of dendritic membrane receptors [49,51]. In axons, Rab7 also localizes to late endosomes and autophagic vesicles, which progressively increase degradative capacity as they localize more proximally [52,53,54,55,56], though retrograde Rab7 late endosome and autophagosome maturation progress independently of one another [57]. Many studies in numerous model systems have characterized the trafficking dynamics of axonal Rab7-positive endosomes and demonstrated that they undergo long-range trafficking within axons, often with retrograde bias, which coincides with their endosomal and autophagosomal maturation [48,54,58,59,60,61,62]. Notably, Rab7-mediated retrograde traffic is also seen within sensory neurons, where Rab7 co-traffics with distally internalized axonal neurotrophin receptors [63]. These are the neurons primarily affected in CMT2B patients.

Neurotrophin receptors, like TrkA with its ligand NGF, promote neuronal survival and proper circuit formation [64,65,66]. Target-organ-secreted neurotrophins are recognized by Trk receptors on distal axons, which together are internalized into an endosome, termed a signaling endosome, and transported retrogradely through axons over exceptionally long distances to potentiate somatic signaling [67]. There is evidence to support that long distance, retrograde signaling endosomes are Rab7-positive late endosomes [60,63,68]. However, Rab5-positive early endosomes were also demonstrated to traffic TrkA [69], and a small subset of TrkA is found in Rab11-positive recycling endosomes [70]. The exact molecular makeup of signaling endosomes could indeed direct their fate since they recruit different downstream effectors that influence their longevity and location and, thus, the strength, duration, and locale of TrkA-mediated signaling cascades [71]. Thus, the signaling endosome comes in several “flavors”, which determine the path that Trk receptors take, but, ultimately, the duration and termination of the signal are dependent on Rab7 function upstream of lysosomes [68]. For instance, in NGF-stimulated rat pheochromocytoma (PC12) cells, TrkA increasingly colocalizes with Rab7, and the overexpression of a dominant-negative, GDP-bound Rab7 (Rab7^DN^) interferes with the degradation of TrkA, prolongs signaling, and promotes subsequent neurite outgrowth [21]. Similarly, the knockdown of Rab7 leads to the retention of TrkA in enlarged Rab5-positive endosomes in PC12 cells [60]. Rab7 knockout abrogates the arrival of TrkA endosomes in the soma of sympathetic neurons grown in cell body and axon compartmentalized cultures [68] and, thus, leads to the loss of survival signaling in the soma. Local signaling from early endosomes in the distal axon might still occur normally or even be prolonged in this experimental paradigm.

## 4. Receptor Tyrosine Kinase Signaling in Charcot–Marie–Tooth 2B Models

Since Rab7 critically regulates the trafficking of signaling receptors, it is reasonable to ask if CMT2B patient mutations alter Rab7-mediated neuronal tyrosine receptor kinase trafficking and signaling. Perhaps surprisingly, the answer to this question has not been so clear cut, as studies investigating the impact of CMT2B mutations on tyrosine kinase receptor signaling are often contradictory. Most work has focused on two tyrosine kinase receptors: EGFR in non-neuronal models and TrkA in peripheral neuron models of CMT2B. Early studies in HeLa cells overexpressing CMT2B mutant Rab7 proteins (L129F, K157N, N161T, V162M) found that EGF/EGFR degradation is similar to that of wild-type cells [33,34]. In further support of this, the knockdown of Rab7 with siRNA led to slowed EGF/EGFR degradation but could be completely rescued by subsequent overexpression of the CMT2B Rab7 mutants [33,34]. An additional study using similar experimental approaches in HeLa cells found that there was an initial delay in EGFR degradation and failure of loaded EGF to traffic out of EEA1-positive early endosomes when Rab7 was downregulated [72]. Interestingly, downstream signaling molecules like pERK levels were retained at high levels for longer and failed to translocate from the cytosol to the nucleus. In this study, Rab7^N161T^ was insufficient to rescue EGFR degradation delays caused by siRNA-mediated Rab7 knockdown, though other mutants were not tested [72]. Differences between these two studies might be due to the different time points measured—by assessing the latest time point used by each of these groups, one might come to the same conclusion that overall EGFR degradation is not impacted in CMT2B overexpression or re-expression models, but the kinetics are slowed. 

The interpretation of these initial studies is complicated by the fact that they had to rely on overexpression or on knockdown coupled to re-expression of Rab7 mutants, which might not recapitulate the levels of Rab7 found in patient cells. However, in line with the conclusions of Basuray et al. 2013, a later study assayed whole cell lysates from dermal fibroblasts and a sural nerve biopsy of a Rab7^K126R^ patient and revealed higher steady-state levels of EGFR plus increased colocalization of loaded EGF with EEA1-positive late endosomes, suggesting that Rab7^K126R^ did not support normal EGF/EGFR degradation [15]. Confusingly, similar studies using Rab7^V162M^ patient fibroblasts demonstrated the opposite, that EGFR degradation was increased and EGF increasingly colocalized with LAMP1-positive lysosomes, leading to decreased levels of signaling molecules like pERK1/2 [73]. These more recent physiologic model systems (i.e., patient tissue) are an important step in avoiding the overexpression complications from early studies. Surprisingly though, different patient alleles from patient-derived cells have shown differences in EGF/EGFR fates. These studies are, thus, beginning to demonstrate that there are functional differences between different CMT2B Rab7 alleles with respect to alterations in receptor trafficking. It is surprising that several CMT2B-associated Rab7 mutants affect EGFR trafficking in opposite directions since they are thought to share common underlying deficits in nucleotide binding. Notably, several Rab7 CMT2B alleles (L129F, K157N, V162M), but not N161T, show slowed fluorescence recovery after photobleaching, indicative of hyperstabilization on endosomes [32,74]. It is clear that our understanding of the molecular defects shared or distinct among the different CMT2B Rab7 alleles is still incomplete.

The data regarding CMT2B mutant Rab7 impacts on NGF-TrkA signaling are more relevant to peripheral nervous system disease but equally conflicting. Importantly, Rab7 can be co-immunoprecipitated with TrkA, and this is not impacted by CMT2B mutants [74]. Studies, for the most part, have found that TrkA-dependent neurite outgrowth is deficient in the following model systems: CMT2B overexpression mutant PC12 (L129F, K157N, N161T, V162M), mouse neuro-2a (N2A) (K126R, L129F, K157N, N161T, V162M), N1E-115 (L129F, K157N, N161T, V162M), patient-derived iPSCs (V162M), and cultured dorsal root ganglia (DRGs) (L129F, K157N, N161T, V162M) [15,60,73,75,76,77]. This is perplexing given that nervous system development in patients appears to proceed normally (i.e., disease onset is in the second or third decade of life). Consequently, it has been widely suggested that defective neurite outgrowth in CMT2B is primarily relevant in axonal regeneration following injury. One study in zebrafish did demonstrate both central and peripheral axon branching deficits at later stages of development. However, the initial stages of axon branching and outgrowth were intact, again suggesting that the neurotrophin-dependent maintenance of healthy axons is a predominant issue [78,79]. How exactly TrkA signaling is dysregulated, leading to defective neurite growth or maintenance, is not exactly clear. For example, in one study, CMT2B mutant (L129F, K157N, N161T, V162M) overexpression in PC12 cells had higher pTrkA and pERK levels following NGF stimulation and retention of pERK1/2 in the cytosol [74]. In contrast, other studies demonstrated that overexpression in PC12 (L129F, K157N, N161T, V162M) [60] and primary iPSC-derived Rab7^V162M^ mutant sensory neurons [73] had decreased pERK1/2 levels following NGF stimulation.

**Figure 3:**
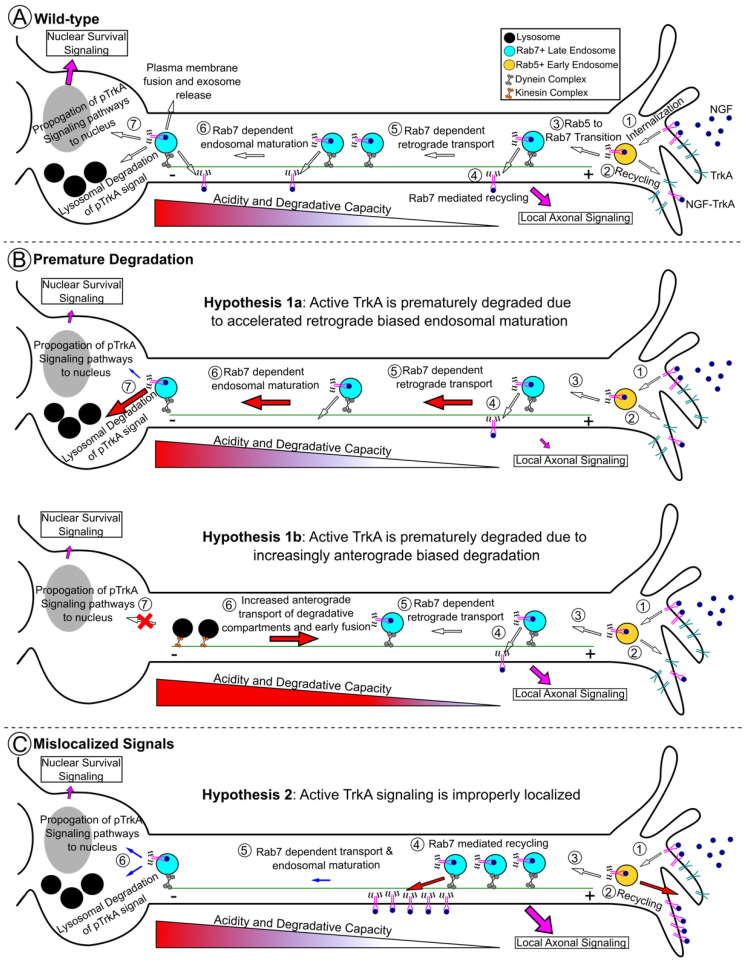
The prevailing hypotheses for how Rab7^CMT2B^ causes disease involve dysregulation of TrkA signaling. (**A**) Schematic of TrkA signaling in a healthy, wild-type neuron. (1) In the distal axon, surface TrkA binds to its ligand NGF secreted by the target organ. Active NGF-TrkA complexes are internalized via endocytosis and arrive at Rab5+ early endosomes (yellow). Active TrkA in an endosome is termed a signaling endosome. (2) TrkA, either unbound or active, that arrives in the early endosome can be recycled to recycling endosomes or back to the plasma membrane, which contributes to sustained local TrkA signaling. (3) Early endosomes mature into late endosomes via Rab conversion from Rab5 to Rab7. (4) Distal Rab7-positive late/signaling endosomes can further recycle TrkA to recycling compartments or the plasma membrane, contributing further to local axonal signaling. (5) Rab7 effector functions, such as RILP-dynein-mediated motility, move signaling endosomes retrograde toward the soma. Further recycling of TrkA might occur as transport proceeds. (6) As signaling endosomes move proximally, Rab7 further contributes to endosomal maturation of signaling endosomes, including increasing degradative capacity and acidification, making them more amenable to TrkA degradation. (7) Fully mature, somatic Rab7-positive signaling endosomes can be degraded in the lysosome (signal termination), propagate signaling cascades to nucleus (survival signaling), or fuse with the plasma membrane and release contents as exosomes. Together, distal local signaling and somatic nuclear signaling contribute to neuronal health. (**B**) Schematic demonstrating the hypotheses that TrkA is prematurely degraded in CMT2B. Top: Accelerated retrograde transport by RILP-dynein and increased fusion with lysosomes depletes both distal and nuclear TrkA signaling leading to neuronal dysfunction. Bottom: Accelerated anterograde transport of degradative lysosomes prematurely degrades TrkA signals within the axon, leading to decreased nuclear signaling but sustained distal signaling. (**C**) Schematic demonstrating the hypothesis that TrkA is mislocalized in CMT2B. Excessive Rab7-dependent TrkA retrieval or failed retrograde transport and endosomal maturation lead to excess local signaling in the absence of nuclear survival signaling.

In the end, despite discrepancies, both EGFR and TrkA data in CMT2B models suggest that there is dysregulation of the trafficking and/or degradation of these receptors. The most coherent explanation of all the data is that Rab7 mutations within peripheral neurons lead to premature degradation of TrkA and, thus, premature the termination of pTrkA signals (Figure 3A,B) and/or that prolonged TrkA signaling in the wrong location, leading to defective neurite behavior (Figure 3A,C). Ultimately, it is currently not clear how all the existing data can be reconciled into a definitive explanation (see also [27,31,35]). More broadly, these data speak for the necessity for all signaling molecules to be present at the right levels (i.e., not degraded) but also in the correct location (i.e., TrkA cannot be stuck in the axon or dendrites; TrkA must be on the surface of endosomes, not on intraluminal vesicles; pERK must reach the nucleus) in order for normal downstream functions, like neurite outgrowth and nuclear transcription-driven survival, to take place. 

## 5. Rab7 Effectors and Physiologic TrkA Trafficking and Degradation 

In order to achieve appropriate NGF signaling, the cell must properly generate and traffic signaling endosomes to the right locations and then degrade TrkA in the right time frame. Rab7-positive TrkA-containing endosomes must rely on Rab7-effector functions to accomplish a number of tasks, which have to occur sequentially. Rab7 effectors bind to Rab7-GTP to control the following: (1) proper motility and positioning of late endosomes, signaling endosomes, and lysosomes (accomplished by the dynein adaptor Rab-interacting lysosomal protein (RILP) and cholesterol-sensitive protein ORP1L), (2) the correct pH (through action of the endosomal vacuolar-ATPase) to activate lysosomal hydrolases, (3) trafficking of newly synthesized lysosomal hydrolases to lysosomes (mediated by mannose-6-phosphate receptors and recycling complexes like retromer), and (4) fusion of late endosomes (through the multimeric HOPS complex) (Figure 2). In this section, we review the key physiologic findings pertaining to these Rab7 effector interactions that are necessary to degrade prototypical cargo, including neurotrophin receptors. 

### 5.1. Rab-Interacting Lysosomal Protein (RILP) and Oxysterol-Related Binding Protein 1 L (ORP1L): Control over Rab7 Motility and Positioning 

Rab7 endosomes move along microtubule tracks via the activity of kinesin and dynein motors. Rab7 interacts with motors through adaptor proteins. The cytosolic adaptor protein RILP links Rab7 with dynein motors [80,81]. In axons, in which microtubule polarity is uniformly plus end out, this means that the predominant retrograde motility observed for Rab7 late endosomes and autophagic vesicles towards the more degradative soma could be accomplished through the Rab7–RILP–dynein complex. In neurons, RILP was suggested to be necessary for this function [55,68]. Recently, however, the two RILP-targeted shRNAs used in these earlier studies appear to have a neuronal-specific off-target phenotype that resulted in the depletion of Golgi staining, in addition to potential on-target effects [82]. Further, knockdown of RILP using a siRNA only minimally affected retrograde motility in the proximal axon and had no effect on medial or distal axonal traffic [83]. Thus, the exact role of RILP in axonal retrograde transport is still under investigation. 

The motility of Rab7 late endosomes via RILP is also regulated by additional features of late endosomes, namely the lipid composition, which affects the recruitment of additional Rab7 effectors. Interestingly, it appears that these interactions are initiated at and regulated by endoplasmic reticulum (ER)–endosome contact sites. ER–organelle contact sites are gaining increasing attention in cell biology and are critical for normal organelle function [84]. The oxysterol-binding protein (OSBP) family binds and mediates the transfer of lipids, like cholesterol, between ER and other intracellular compartments [85]. Oxysterol-binding protein (OSBP)-related protein 1 L (ORP1L) was identified as a Rab7 interacting protein [86]. It was found that in addition to RILP, the proper activation of dynein and subsequent positioning of late endosomes require a tripartite complex with ORP1L in a cholesterol-dependent manner [86,87,88]. The tripartite complex mediates late endosome contact sites with the ER, which ultimately influences endosomal positioning [88,89]. This is dependent on ORP1L responses to cholesterol availability. As evidence of this, excess cholesterol, such as in diseases like Niemann Pick type C [90], drives late endosomal positioning to perinuclear regions, whereas cholesterol depletion leads to late endosomal accumulation at microtubule plus ends [91]. ORP1L is also required for the resolution of phagosomes, suggesting that the degradation of internalized cargo is, in part, dependent on its function [92]. How ORP1L contributes to the control of late endosomes within the complexity of neuronal space, however, has not been elucidated. Given our present understanding of ORP1L function, alterations in Rab7-ORP1L interactions and cholesterol metabolism have the potential to disrupt dynein activation via RILP and, ultimately, endosome positioning, which could negatively impact TrkA trafficking or degradation.

### 5.2. V-ATPase

As endosomes mature from early to late endosomes, then to lysosomes, the intraluminal pH becomes increasingly more acidic. Endo/lysosomal pH is, in large part, established through the activity of the endosome-localized vacuolar H^+^-ATPase (V-ATPase), which is regulated by the assembly of a cytosolic ATPase-containing V_1_ complex with a membrane-embedded proton-translocating V_0_ domain [93]. Correct endosomal/lysosomal pH is critical for functions, such as cargo degradation and ligand receptor dissociation [94,95]. Rab7 has been implicated in V-ATPase function and endosomal acidification through direct interaction with the V_0_a_3_ subunit in secretory lysosome trafficking [96] and by forming a ternary complex with RILP and the cytosolic V_1_G_1_ subunit of the V_1_ complex to regulate V_1_G_1_ availability [97]. The data from the latter study suggest that RILP is responsible for the correct endosomal–lysosomal localization of V_1_G_1_ and, thus, involved in regulating acidification [97]. Fitting this notion, acidification was positively correlated with the level of Rab7 on endosomes, and the depletion of RILP by siRNA led to global increases in endosomal/lysosomal pH [95,98]. Acidification is also important for neurotrophin signaling, as treatment with the V-ATPase inhibitor Bafilomycin-A1 (BafA1) results in failed recycling of TrkA to the plasma membrane and accumulation within intracellular compartments [99]. Following NGF stimulation, BafA1-treated cells have lower levels of pAkt and pERK, suggestive of deficient TrkA signaling [99]. Conversely, hyper-acidification of endosomes leads to less neurotrophin signaling [100]. Thus, degradation of Trk receptors and NGF-TrkA dissociation events are, in part, dependent on both Rab7 function and V-ATPase-mediated endosomal acidification to the correct pH. Furthermore, acidification might also impact endosomal localization along axons since, in fibroblasts, less-acidified endosomes/lysosomes localize more peripherally [98]. 

### 5.3. Vps35: Connections to Both TrkA Sorting and Degradation

The degradation of any cargo like TrkA requires functional lysosomes, which depend on the proper trafficking of degradative hydrolases, such as cathepsins. Cathepsins are trafficked via specialized pathway(s) that involve binding of a mannose-6-phosphate moiety in the trans-Golgi network (TGN) by the mannose-6-phosphate receptor (M6PR) and trafficking to late endosomes, where M6PR dissociates from its cargo and recycles to the TGN [101]. This retrieval pathway is, in part, dependent on the activity of retromer [102,103,104]. If retrieval is defective, M6PR becomes stuck in endosomes and fails to continue to deliver cathepsins to the late endosome/lysosome. To what degree M6PR trafficking and lysosomal biogenesis in particular cell types depend on the activity of retromer is actively debated [104,105]. 

Retromer is composed of a trimeric core complex involved in cargo sorting (Vps26/29/35) and heterodimeric SNX-BAR family proteins, which facilitates membrane tubulation. The core retromer complex subunit Vps35 is a Rab7 effector protein [106,107,108]. Vps35 interacts directly with active GTP-bound Rab7 [106]. Increased GTP-Rab7 levels following the depletion of the Rab7 GAP TBC1D5 increase membrane recruitment of retromer, whereas the overexpression of the GAP inhibits membrane localization [107,109,110]. Rab7 knockdown or disruption of Rab7-Vps35 interaction leads to the subsequent loss of Vps26 membrane localization, suggesting that Vps35-Rab7 is a key component of retromer assembly [107,108]. Knockout of Vps35 leads to clear changes in lysosomal morphology and degradative capability [103,111,112]. Vps35 is likely especially critical in neurons, as it is a genetic locus for familial Parkinson’s disease, which has been discussed extensively elsewhere [113,114,115]. Recent omics data validate this importance, as Vps35 knockout neuroglioma cells exhibit a wide breadth of proteomic and transcriptomic changes pertaining to endosomal function [116]. However, what role Vps35 plays in neurotrophin trafficking is not fully understood. At a minimum, it has been demonstrated that a subset of Rab7+TrkA+ signaling endosomes are positive for Vps35 [68]. 

### 5.4. Homotypic Fusion and Protein Sorting (HOPS) Complex for Tethering and Fusion 

The multimeric HOPS complex was identified as the Rab7 effector machinery that facilitates membrane tethering and fusion between late endosomes and the vacuole in yeast [117,118,119]. HOPS subunits Vps39 and Vps41 can bind Rab7 in pulldown experiments in vitro [119,120]. In liposome assays, fusion required Rab7 on both opposing membranes and the presence of HOPS [121]. Even though HOPS subunits are able to bind Rab7, evidence that HOPS is a direct Rab7 effector protein in mammalian cells is lacking [28]. Instead, there is evidence that other small GTPases, such as Arl8b or Rab2, mediate the recruitment of HOPS for fusion in mammalian systems [122,123]. Furthermore, Rab2 and/or Arl8b may instead facilitate the inactivation of Rab7 in a HOPS-dependent manner [124]. Nevertheless, the HOPS complex can simultaneously bind Rab7 and RILP and form Rab7-HOPS-RILP complexes, which are likely necessary for lysosomal fusion events in mammalian cells [125,126,127,128]. It is, thus, possible that RILP-Rab7 recruits HOPS machinery onto late endosomal/lysosomal membranes where they are handed off to Rab2 or Arl8b, and then Rab7 is inactivated prior to actual fusion. The details of these cascades are still not fully elucidated nor are the specific implications for HOPS in neurotrophin trafficking and degradation. 

## 6. A Novel Notion of Effector Balance and Ordering: Is this Disrupted in Disease?

To this point, we have discussed the changes to TrkA signaling cascades in CMT2B and physiologic interactions of Rab7 with various effectors. In the following sections, we will build on the notion that CMT2B is a product of dysregulation of TrkA signaling due to degradative impairments and/or signaling in the wrong location (Figure 3) and ask how disease-related changes in specific Rab7 effector interactions may drive either of these outcomes. 

Notably, however, the experiments conducted up to this point elucidate changes in Rab7 CMT2B mutant behavior with regard to a single effector. This is clearly an important first step to provide foundational evidence of changes in Rab7 function in CMT2B disease. It is, however, a great simplification of the cytosolic milieu of multiple effectors that GTP-bound Rab7 may encounter. At any given moment, which effector does active Rab7 choose to bind? In our minds, this minimally depends on the affinity of Rab7 for a particular effector and the absolute abundance of any given effector versus another. In a hypothetical situation of active Rab7 with equal affinity for two different effectors, but one at a high concentration and one at a low concentration, we would expect the effector present in excess to be bound by more GTP-Rab7, simply as a consequence of mass action. These are all important notions, as it has long been proposed that CMT2B is a disease of quantitative, rather than qualitative, Rab7 effector changes [32]. In addition, the spatiotemporal ordering of Rab7 effector cascades along lengthy axons might be changed in CMT2B Rab7 alleles, altering where and when active TrkA is signaling, which needs to be considered. Therefore, in addition to reviewing known data regarding how Rab7 effectors are changed in CMT2B and what this means for TrkA, our discussion will bear differential Rab7 affinity, effector abundance, and neuronal spatial complexity in mind. 

### 6.1. RILP in CMT2B: A Case for Effector Abundance?

**Figure 4:**
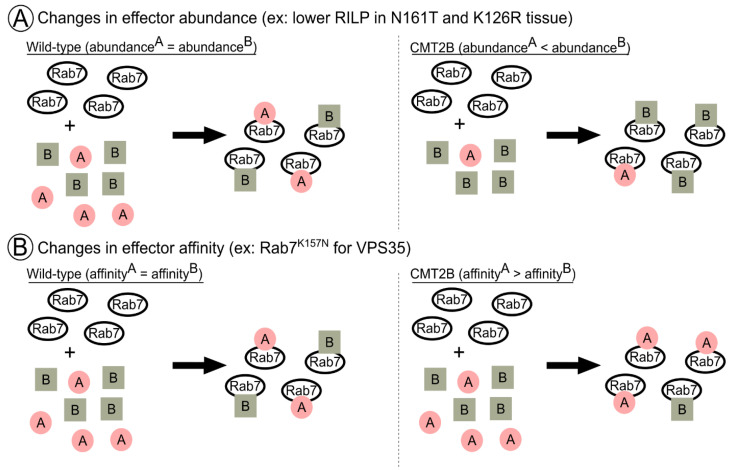
Effector abundance and affinity affect Rab7 functional balance. (**A**) Changes in absolute abundance of an effector protein can alter Rab7 function balance. Left: In control scenarios where effectors A and B are in equal abundances, under the assumption that affinities are also equivalent, Rab7 will bind A and B in equal proportions. Right: In CMT2B, where effector abundance is altered (e.g., lower RILP (represented by A) in N161T and K126R patient tissue), but affinities are assumed to remain equivalent, Rab7 will bind effector B in greater proportion to effector A (RILP). (**B**) Changes in Rab7 affinity for particular effectors can alter Rab7 function balance. Left: In control scenarios where Rab7 has equal affinity for equally abundant effectors A and B, Rab7 will bind A and B in equal proportions. Right: In CMT2B, where Rab7 has altered affinity for particular effectors (e.g., decreased affinity for VPS35 (represented by B) in K157N), under the assumption of equal abundance, Rab7 will bind effector B (VPS35) in lesser proportion to effector A.

The overexpression of CMT2B mutant Rab7 proteins in HeLa cells did not alter RILP expression [33], and co-immunoprecipitations of overexpressed Rab7 mutants and RILP demonstrated equal and preserved interactions as compared to wild type [32]. However, sural nerve biopsies from the Rab7^N161T^ mutant proband stained for RILP demonstrated the loss of RILP protein levels compared to healthy controls [12]. Similarly, whole cell lysates from sural nerve biopsies of the Rab7^K126R^ proband also showed reduced RILP levels [15]. Thus, in patient peripheral nervous system tissues, RILP expression appears to be decreased but, most likely, the affinity of CMT2B Rab7 alleles for RILP is not changed (Figure 4A). It is not known why levels of Rab7 effectors (such as RILP) would be decreased in CMT2B. It is also not known if all CMT2B alleles equally affect expression levels of RILP nor if other (or all) Rab7 effectors also change levels in CMT2B. Regardless, lower levels of RILP could have profound impacts on dynein-mediated retrograde trafficking of TrkA containing endosomes by having less RILP available to hook endosomes to motors. Considering that RILP is also a part of tripartite Rab7 effector complexes (like the V-ATPase and HOPS; Figure 2), the lower levels of RILP could also affect the function of these complexes. This also raises the question of whether certain complexes (such as Vps35/retromer) would then become favored in a low-RILP environment and in what proportion. Changed Rab7 effector complex distribution due to low RILP may dysregulate TrkA endosome behavior because both Rab7-RILP function is lost and Rab7 complexes with another effector are increased. 

What is the experimental evidence for alterations in RILP behavior, and what are its effects on intracellular trafficking of Rab7 and TrkA in CMT2B? There are data to support both the TrkA degradation (Figure 3B) and TrkA mislocalization hypotheses (Figure 3C). Multiple studies across model systems have concluded that CMT2B mutations result in increased speed and processivity of Rab7-positive endosomes in the axon, though they disagree on the impacts on movement directionality (anterograde or retrograde) [60,79,129]. Faster retrograde motility could prematurely deplete signaling endosomes from distal axons and shorten the local signaling cascades required for axon growth. In addition, faster retrograde motility of Rab7 endosomes could lead to the premature degradation of TrkA cargo within the soma and shorten somatic signaling cascades or, alternatively, an excess of somatically arriving signaling endosomes, which signal for longer (Figure 3B). Which of these outcomes is observed might depend on the individual CMT2B allele and its particular effector binding affinity and particular changes in effector abundance. 

Similarly, faster anterograde motility of degradative lysosomes and fusion with TrkA carriers could also prematurely degrade the signal within the axon, preventing the signal from reaching the soma (Figure 3B). Zhang et al. 2013 found increased anterograde motility of Rab7^N161T^, though they did not assess other mutants. Notably, this is also one of the mutants in which we have patient data on RILP levels. It is reasonable that the decreased abundance of RILP in this mutant leads to a shift in the bias of anterograde to retrograde transport given the requirement of RILP for retrograde motility. 

In contrast to the above-cited studies, in a zebrafish transgenic model with in vivo live imaging more closely mirroring physiologic protein levels, Rab7^L129F^ and Rab7^K157N^ endosomes had decreased velocities, and Rab7^N161T^ and Rab7^V162M^ endosomes exhibited a stationary bias [78]. These findings are consistent with the pathological loss of RILP observed in patient samples and support the notion that a decreased abundance of RILP impacts Rab7 effector function. These data also support the hypothesis that active TrkA reaches the soma and encounters degradative compartments more slowly in CMT2B neurons, possibly resulting in extended signaling in the axon and decreased signaling in the soma (Figure 3C). 

Nevertheless, the impacts on Rab7 endosome motility in CMT2B are debated. With the advent of newly developed transgenic models [26], advances in in vivo imaging, and increasing availability of patient-derived iPSCs, better studies can be carried out to more definitively elucidate the impacts on Rab7 and TrkA motility in relevant heterozygote models and relevant cell types. Side-by-side comparisons of all mutants in these most physiologic models, particularly in microfluidic devices to separate distal axon and somatic signaling effects, will help clear up the discrepancies between previous overexpressed in vitro studies and in vivo studies and determine the impact and differences between mutants for axonal traffic. 

### 6.2. VPS35 in CMT2B: A Case for Effector Affinity?

Multiple studies have determined that some CMT2B Rab7 mutants have altered interactions with Vps35. Overexpressed Rab7^K157N^ immunoprecipitated significantly less Vps35 than Rab7WT overexpression cells [107]. Rab7^L129F^ and Rab7^N161T^ mutants were also tested, and though they had mild decreases in interaction with Vps35, they were not significantly changed [107]. Rab7^K157N^ also could not rescue retromer-component membrane localization to the same degree as Rab7^WT/L129F/N161T^ [107]. Further, in liposome sedimentation assays, a combination of SNX3 and Rab7^WT^ was shown to be sufficient to drive retromer association with membranes, versus either SNX3 or Rab7^WT^ alone. However, Rab7^K157N^ could not drive the same recruitment, even in the presence of SNX3 [130]. The lack of Vps35 immunoprecipitation by Rab7^K157N^, as compared to other mutants in an identical experimental paradigm where Rab7 and Vps35 levels were controlled, is evidence of a change in affinity for Vps35 by this particular mutation (Figure 4B). Whether or not this holds true in vivo where abundances, both global and locally, can vary remains to be seen. The consequences of deficient Rab7^K157N^-Vps35 interactions on TrkA or lysosomal enzyme trafficking, however, have also not been elucidated. One could speculate that reduced Rab7^K157N^-Vps35 interactions would result in failed recycling of TrkA receptors, resulting in degradation rather than prolonging the duration of an active TrkA receptor. In contrast, data from Rab7^V162M^ patient fibroblasts and iPSC-derived sensory neurons show alterations in retromer-dependent processes as they contain increased levels of M6PR and mature cathepsins [73]. Similarly, Rab7^K126R^ patient fibroblasts [131] had more mature cathepsin and degradation of the sensor DQ-BSA, suggesting increased degradation. However, we do not currently know the true impact of these particular mutations on Vps35-Rab7 interactions [107] nor the impact of Rab7 mutations on local or somatic signaling. 

In addition to Vps35 core retromer complex sorting activities, retromer-associated SNX-BAR proteins promote membrane tubulation. In Markworth et al. 2021 [77], the authors investigated the relationship between Rab7+ tubule formation and active TrkA. The stimulation of DRGs and mouse embryonic fibroblasts (MEFs) with NGF resulted in active TrkA-containing Rab7 tubules. In MEFs overexpressing Rab7^K157N^ and Rab7^V162M^, there was no increase in tubulation following NGF stimulation and, subsequently, lower levels of active TrkA. In Rab7^L129F^ and Rab7^N161T^, tubulation was increased at baseline and could not be further increased by NGF addition, resulting in persistently high active TrkA levels. The exact dependence on Vps35 or SNX family proteins was not established in this study, but it is evident that TrkA incorporation into tubules is key to controlling signaling fates and that the various mutant alleles impact this process differently. Together, similar to EGFR degradation and Rab7 motility studies, it is clearly evident that different CMT2B alleles show clear differences in retromer-related enzyme trafficking and tubulation phenotypes that may be tied to changes in affinity. Since all CMT2B alleles appear to be fast cycling in biochemical assays, the definitive structural basis for these differences is not clear.

### 6.3. Spatial Ordering of Rab7 Effectors along the TrkA Trafficking Route 

**Figure 5:**
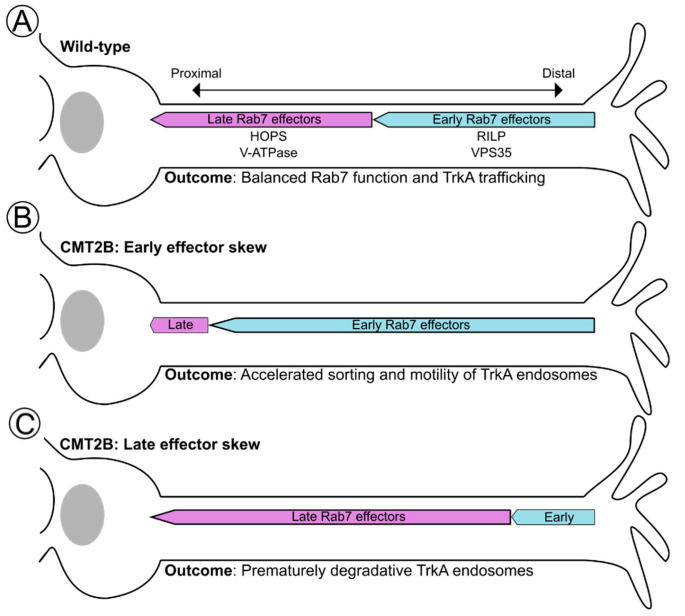
Changes in Rab7 effector binding within neuronal space impact TrkA signaling. (**A**) Under wild-type conditions, Rab7 effector functions are grouped into early, distal effectors (RILP, Vps35), which position active TrkA in the correct location, and late, somatic effectors (HOPS, V-ATPase), which prime endosomes for TrkA degradation and signal termination. Balanced distal versus somatic effector binding and functions ensure proper TrkA trafficking and both local and nuclear signaling. (**B**) Possible early effector skew in CMT2B leads to more RILP and/or VPS35 function across greater neuronal space. As a result, excess motility and/or sorting of TrkA receptors contributes to either premature degradation or improper localization of TrkA signals. These scenarios would have negative impacts on the sustainment of proper nuclear and local signaling. (**C**) Possible late effector skew in CMT2B leads to more HOPS and/or V-ATPase function across greater neuronal space, contributing to degradative endosomes or lysosomes further down the axon and leading to early termination of TrkA signaling. Early termination of TrkA signals within the axon would lead to a loss of necessary nuclear survival signaling.

Consider again an active, internalized TrkA receptor, which must travel from the distal axon to the soma and undergo multiple endosomal maturation steps to potentiate signaling or be degraded. As we have discussed, these steps, including motility, sorting, acidification, and fusion, are Rab7-dependent. They also exist along a distal to proximal gradient within neuronal processes. TrkA signaling must also exist in a gradient in order to support local effects at distal axons, like axonal growth and maintenance, and somatic global effects like survival. This then raises questions: which effectors are needed first, and is there particular ordering to Rab7 effector cascades in space that impacts TrkA signaling? We speculate that the Rab7 effector functions can be grouped into early and late effectors (Figure 5). Early effectors, like RILP and Vps35, can potentiate the long-range movement and recycling of receptors and, thus, contribute to correct distributions of TrkA receptor complexes. Late effectors, like HOPS and the V-ATPase, which mediate lysosomal fusion and acidification, respectively, would advance TrkA and other cargos towards degradation. Thus, within the large space of the axon, early effector functions would occur more distally, allowing distal signaling to occur as well as promoting retrograde transport towards the soma, whereas late effector functions would occur more proximally in the axon and in the soma to promote fusion with somatic lysosomes. There is a body of evidence just beginning to support this notion. For instance, a recent study found that there is an ordering to Rab effector cascades. Specifically, Rab7 is found with RILP first on earlier endosomal compartments and only later engages with HOPS on more mature compartments to mediate lysosomal fusion [123]. Furthermore, pH-neutralized distal TrkA carriers are still able to retrogradely traffic from the distal axon in compartmentalized chambers all the way to the soma [132], suggesting that acidification is not a prerequisite for motility, and Rab7-RILP-dynein complexes likely precede that of V-ATPase complexes. This is further supported by the long-standing notion that acidification progresses as a compartment moves retrograde and that the most acidic compartments are somatically localized [133]. 

What are the implications for distal-to-proximal axon Rab7 effector cascades with respect to CMT2B Rab7 alleles that may change effector choice? Progressing from the most distal to most proximal, excessive engagement of an early effector like Vps35 would result in excessive active TrkA distally. With the consideration that local TrkA signaling may potentiate axonal growth, while somatic signaling would support survival, excessive TrkA sorting distally would diminish neuronal survival. A similar outcome could be achieved in settings of low-RILP-driven motility (as in N161T and K126R mutants) [12,15], where TrkA carriers become stalled distally (Figure 3B,C and Figure 5B). Conversely, high engagement with RILP or loss of interaction with Vps35 (as in K157N [107,130]) would deplete local axonal growth signals and could support either excessive somatic degradation or somatic survival signaling of active TrkA. 

Alternatively, excessive or premature engagement of a late effector like HOPS or the V-ATPase on retrograde Rab7+TrkA+ carriers and an acidified compartment, for example, might result in early degradation of the signal within the axon, in line with the model proposed in Zhang 2013 (Figure 3B and Figure 5B). Multiple studies report that a small number of acidified and degradative LAMP1+ compartments, i.e., lysosomes, move anterogradely and are present in axons [134], which could potentiate such degradation. However, most LAMP1+ compartments in axons are not highly acidic or degradative [53] or are Golgi carriers [54], and the premature fusion of anterograde degradative compartments with retrograde TrkA carriers has not been directly observed. The disruption of HOPS-mediated fusion or V-ATPase-mediated acidification would support too much signaling, both somatically and distally. As evidence of this, treatment with the V-ATPase inhibitor Bafilomycin-A1 (BafA1) results in the accumulation of TrkA within intracellular compartments, which may be signaling competent [99]. Further, NGF- and BafA1- or NH4Cl-treated distal axons still traffic FLAG-tagged TrkA to the cell body following NGF stimulation, but the spatial gradient of NGF-TrkA signaling output is shifted from in the cell body distally into the axon [132]. 

Together, effector affinity, abundance, and ordering likely all contribute to normal Rab7 function and, ultimately, endosomal maturation and TrkA signaling gradients within neuronal space. However, we know remarkably little about these processes for wild-type Rab7 and even less so for CMT2B mutants. It is unclear what balance between affinity and abundance changes exists in CMT2B disease and, ultimately, how this affects the effector ordering within the axon. However, it is clear that the balance of all of these factors is necessary for faithful trafficking and degradation of TrkA, and without such distal local signaling, somatic signaling and degradation become disrupted. 

## 7. Other Rab7 Hypotheses: It Is Not all about TrkA

Even though we have discussed Rab7 within the hypothesis that TrkA signaling is dysregulated in CMT2B, this is not the only current possible contributing mechanism underlying CMT2B as other data support defects in additional processes. For instance, CMT2B mutants display dysregulation in autophagy and lipophagy progression, leading to the failure of autophagic clearance and lipid droplet accumulation [131,135,136]. These defects might also lead to the observed axonopathies observed in CMT2B. Rab7 has known roles in autophagy progression [137], which requires orderly Rab7 effector progression, as discussed. These data also provide an interesting future avenue of investigation for a lipid-centric hypothesis of CMT2B, given that the CMT2B mutant Rab7 increasingly binds both ORP1L and the lipid transfer protein Vps13C [32,138], though it is unclear whether this is due to changes in abundance or affinity. Mutant cells also have excessive lipid droplets and broad changes in their lipidome [131,136]. Increased Rab7-ORP1L interactions could have profound impacts on endosome motility, given their involvement in a tripartite complex with RILP, though this remains to be explored in the setting of disease. The literature also supports a role for cholesterol-sensitive ER–endosome contact sites in mediating neurite health and outgrowth, as the loss of other Rab7-interacting ER-resident proteins like protrudin negatively affect axonal growth and the entry of anterograde traffic into the axon [139,140,141]. In Rab7^V162M^ patient fibroblasts, the proportion of neutral lipids made up by cholesterol is decreased [136]. In settings of low cholesterol, ER-late endosome contact sites are increasingly formed; ORP1L is not engaged with RILP-dynein complexes and instead engages with autophagosomes, which is inhibitory to Rab7-mediated minus-end directed transport [88,142]. This would result in distal stalling of TrkA endosomes and, thus, possible signaling in the wrong location. Thus, altered ER–endosome contacts via ORP1L could be impactful for axonal maintenance. Ultimately, the increased ORP1L/Vps13C interactions with Rab7 mutants with concomitant decreases in cholesterol will need to be simultaneously validated within patient tissues and novel knock-in model systems to better understand the contribution of changes in lipid metabolism in CMT2B disease. 

Further, given the specificity of the impact of Rab7 mutations on the peripheral nervous system (PNS), it has long been speculated that there may be a unique PNS Rab7 effector that mediates disease [35]. To this end, it was found that the intermediate filament peripherin, specific to the PNS, more highly interacts with all of the Rab7 CMT2B mutants versus wild-type Rab7 [15,143]. Higher levels of peripherin were also found within Rab7^K126R^ patient dermal punch biopsies [15]. It is thought that Rab7 mutants may impact the balance of soluble to insoluble peripherin, affecting filament formation. Since the roles of neuronal intermediate filaments with regard to endosomal trafficking and the long-term health of neurons are poorly understood, it is not at all clear how changes in Rab7-peripherin interactions in CMT2B might contribute to pathologies [143]. Finally, recent studies have begun to emphasize the impacts of Rab7 on mitochondrial health and how this may become changed in disease, resulting in altered neuronal metabolism. Multiple studies have described both increased retrograde-biased motility and the morphology of mitochondria in settings of CMT2B Rab7 mutants [26,79]. Within a new heterozygous Rab7^V162M^ knock-in model, mitochondrial trafficking deficits were specifically found within the primary peripheral sensory neurons and not in primary hippocampal or cortical neurons [26]. Whether this relates to peripherin expression is not known. Local translation of key mitochondrial proteins is also diminished in mutants [79]. Given that some of the regulation of Rab7 activity takes place in mitochondria [110,144,145], GEF-independent cycling of Rab7 may reciprocally disrupt typical mitochondrial dynamics. In support of this, Rab7^V162M^ mutants displayed prolonged lysosomal–mitochondrial contacts, which ultimately altered inter-mitochondrial tethering dynamics [146].

## 8. Concluding Remarks

In conclusion, the peripheral neuropathy Charcot–Marie–Tooth 2B can largely be attributed to alterations in Rab7 interactions with its many important effectors, as summarized in Table 1. The prevailing hypothesis, among others, has been that dysfunctional Rab7 nucleotide cycling and subsequent alterations in effector interactions disrupt intracellular trafficking and lead to deficits in neuronal survival signals via the NGF-TrkA neurotrophin receptor complex. Since different CMT2B alleles show distinct alterations in Rab7 effector binding, we postulate that, currently, unexplored changes in differential effector affinities and levels as a consequence of mutations may alter the spatial ordering of Rab7 effector interactions along an axon, leading to either premature degradation of TrkA signaling or mislocalization of the signal. With recent advances in better physiologic models, as well as increased access to patient tissues, the field is making significant progress in accurately understanding the underlying causes of the CMT2B, which were previously complicated by non-neuronal overexpression systems. We are also closer to answering some of these more complicated questions regarding Rab7 effector balance and cargo trafficking and how this is altered in disease.

## Figures and Tables

**Table 1:** Summary of Rab7 effector changes in CMT2B.

Effector	Function	Rab7^CMT2B^ Interaction vs. Rab7^WT^	Abundance	Observed Outcomes for CMT2B Alleles Attributable to Effector
RILP	Dynein motor adaptor protein; late endosomal positioning and motility	Equal, in overexpression followed by immunoprecipitation experiments [32,33]	Decreased in N161T and K126R patient tissues [12,15]	-Increased anterograde motility [60] -Increased retrograde motility [79] -Decreased stationary and pausing time [78,79,129] -Increased endosome speeds [60,79] -Decreased endosome speeds [78]
ORP1L	Late endosome positioning and motility (w/RILP); lipid exchange and ER contacts	Increased by IP-MS [L129F, V162M] [32]	Undetermined	-Positioning and motility same as RILP -ER contact sites undetermined
VPS13C	Lipid exchange and ER contact sites	Increased by IP-MS [L129F, V162M] [32]	Undetermined	-Increased lipid droplet abundance [131,136] -Increased cholesterol ester:cholesterol ratios, increased monounsaturated fatty acids, free fatty acids, and total neutral lipids [136]
VPS35	Core retromer complex component; sorting and recycling of membrane receptors	-Preserved [L129F, N161T] -Decreased [K157N] -Undetermined [V162M, K126R] [105]	Undetermined	-Increased M6PR levels [V162M] [73] -Increased mature cathepsins [V162M/K126R] [73,131] -Increased TrkA tubule behavior [L129F/N161T]; no change in TrkA tubule behavior [K157N/V162M] [77]
V-ATPase Subunits	Endosomal and lysosomal acidification (w/RILP)	Undetermined	Undetermined	Undetermined
HOPS Subunits	Endosomal tethering and fusion (w/RILP)	Undetermined (possibly decreased [27])	Undetermined	Undetermined
Peripherin	Peripheral nervous system specific intermediate filament	Increased [15,143]	Increased [15]	-Increased soluble: insoluble peripherin [143] -Undetermined impact on endosome behavior

## Data Availability

Not applicable.
